# Resistance to viral nervous necrosis in European sea bass (*Dicentrarchus labrax* L.): heritability and relationships with body weight, cortisol concentration, and antibody titer

**DOI:** 10.1186/s12711-021-00625-2

**Published:** 2021-04-01

**Authors:** Sara Faggion, Daniela Bertotto, Massimiliano Babbucci, Giulia Dalla Rovere, Rafaella Franch, Mauro Bovolenta, Stanislas Laureau, Francesco Pascoli, Anna Toffan, Luca Bargelloni, Paolo Carnier

**Affiliations:** 1 Department of Comparative Biomedicine and Food Science (BCA), University of Padova, Legnaro, PD Italy; 2 Valle Cà Zuliani Società Agricola Srl, Conselice, RA Italy; 3 Istituto Zooprofilattico Sperimentale Delle Venezie, National Reference Laboratory (NRL) for Diseases of Fish, Mollusk and Crustacean, Legnaro, PD Italy

## Abstract

**Background:**

Susceptibility of European sea bass (*Dicentrarchus labrax* L.) to viral nervous necrosis (VNN) is well-known. Interest towards selective breeding as a tool to enhance genetic resistance in this species has increased sharply due to the major threat represented by VNN for farmed sea bass and limitations concerning specific therapeutical measures. A sea bass experimental population (N = 650) was challenged with nervous necrosis virus (NNV) to investigate genetic variation in VNN mortality. In addition, relationships of this trait with serum cortisol concentration after stress exposure, antibody titer against NNV antigens, and body weight at a fixed age were studied to identify potential indicator traits of VNN resistance.

**Results:**

The estimate of heritability for VNN mortality was moderate and ranged from 0.15 (HPD95%, 95% highest posterior density interval: 0.02, 0.31) to 0.23 (HPD95%: 0.06, 0.47). Heritability estimates for cortisol concentration, antibody titer, and body weight were 0.19 (HPD95%: 0.07, 0.34), 0.36 (HPD95%: 0.16, 0.59) and 0.57 (HPD95%: 0.33, 0.84), respectively. Phenotypic relationships between traits were trivial and not statistically significant, except for the estimated correlation between antibody titer and body weight (0.24). Genetic correlations of mortality with body weight or antibody titer (− 0.39) exhibited a 0.89 probability of being negative. A negligible genetic correlation between mortality and cortisol concentration was detected. Antibody titer was estimated to be positively correlated with body weight (0.49).

**Conclusions:**

Antibody titer against NNV offers the opportunity to use indirect selection to enhance resistance, while the use of cortisol concentration as an indicator trait in breeding programs for VNN resistance is questionable. The estimate of heritability for VNN mortality indicates the feasibility of selective breeding to enhance resistance to NNV and raises attention to the development of genomic prediction tools to simplify testing procedures for selection candidates.

## Background

Disease outbreaks are, in general, severe threats that can interfere with the progress and sustainability of intensive aquaculture systems. Viral nervous necrosis (VNN), also known as viral encephalopathy and retinopathy (VER), affects more than 50 different marine and freshwater fish species. In recent years, nodavirus (or nervous necrosis virus, NNV) infections has had serious consequences, both in terms of animal and economical losses [1]. NNV is a non-enveloped icosahedral virus of the Nodaviridae family, genus *Betanodavirus*. Major damages caused by this virus have been observed in the central nervous system of the fish (brain, spinal cord and retina) [2], with recognizable clinical and behavioural signs (abnormal swimming patterns, lethargy, skin darkening and loss of appetite) [3].

European sea bass (*Dicentrarchus labrax* L.), one of the most common and valuable marine species that is widely cultured in the Mediterranean areas (FEAP Annual report 2017), is one of the species that is recognized as being susceptible to NNV. The severity of NNV outbreaks is strictly related to water temperature, with an optimum between 25 and 30 °C for the genotypic variant that affects the European sea bass, i.e. the red-spotted grouper NNV (RGNNV) [4]. Late spring, summer, and the beginning of autumn are the most affected seasons, depending on water temperature fluctuations. However, mortality due to NNV outbreaks has also been registered during winter at a temperature of 20 °C [5]. Outbreaks of VNN lead to a mortality of 80 to 100% at the larval stage [6] and moderate mortalities have been reported even in advanced juveniles (11–20%) [7]. In survivor fish, NNV infection can become chronic and cause poor growth rates [8].

Chemotherapeutics are not effective for controlling VNN [1]. Different NNV vaccination approaches have been investigated for sea bass, including those based on synthetic peptides of capsid proteins [9], virus-like particles (VLP) [10], inactivated viruses [11], DNA vaccines [12], and recombinant vaccines produced in bacteria [13]. Although some of these vaccination strategies show relatively good results in terms of protection against NNV, only two commercial NNV vaccines are currently available, both based on inactivated RGNNV (Alpha Ject micro® 1Noda, PHARMAQ, and Icthiovac VNN, HIPRA). The type of administration (intraperitoneal injection) and the relatively short duration of protection (maximum 12 months) raise questions about their applicability in the context of aquaculture farming. Moreover, the minimum weight required to inject the vaccine (12 to 15 g) precludes protection of the larval stages, which is when infectivity of the virus is highest.

Recently, attention has been directed to selective breeding as a tool to enhance resistance to VNN because, in general, host resistance plays a key role in hindering pathogen spread or in lowering infection pressure. Interest in selective breeding as a disease prevention action is supported by the magnitude of the additive genetic variation that has been estimated for disease resistance in different aquaculture species. Indeed, significant additive genetic variation for resistance to VNN in sea bass has been reported [14, 15], making selective breeding a possible option to obtain both long-term control of the disease [16] and cumulative and permanent improvement in resistance over generations [1]. For some species, such as salmonids, genetic selection of strains for improved disease resistance is well established and supported by results of commercial selection programs [17, 18].

Indirect approaches that exploit non-trivial genetic relationships of indicator traits with disease resistance may facilitate selective breeding for resistance to pathogens. In fact, if disease resistance is significantly genetically correlated (positively or negatively) with another trait, selection could be performed on the second trait to obtain a correlated response in disease resistance.

Post-stress cortisol level could be an indicator trait for resistance to VNN due to its reliability in terms of stress assessment [19] and presence of substantial additive genetic variation, as evidenced by heritability estimates ranging from 0.08 to 0.33 [20, 21]. The use of cortisol levels in selective breeding has been scarcely investigated and the genetic relationship between this physiological trait and disease resistance is unclear. In sea bass, estimates of the genetic correlation between post-stress cortisol concentration and resistance to VNN are lacking.

Immunological parameters that may be genetically correlated with disease resistance and that show genetic variation could also be of interest. The genetic component of variation in antibody response has received very limited attention in fish species. Two studies on Atlantic salmon [22, 23] reported medium to low estimates of heritability for antibody titer against antigens of *Vibrio anguillarum* and *Vibrio salmonicida*. To date, in sea bass, neither the genetic variation in antibody titer after viral or bacterial infection nor the genetic relationship of antibody titer with disease resistance have been investigated. Thus, the aim of our study was to estimate the genetic parameters of mortality of sea bass to VNN and of candidate indicator traits, such as cortisol concentration after stress exposure, antibody titer against NNV antigens, and body weight at a fixed age.

## Methods

### Production and rearing of experimental fish

The experimental fish were produced in the commercial hatchery of Valle Cà Zuliani Società Agricola srl (Pila di Porto Tolle, Rovigo, Italy) through artificial fertilization, using dams and sires that, on average, were seven and eight years old, respectively. Dams were conditioned to reproduction by modifying the photoperiod and thermoperiod to mimic the natural reproductive season. Sperm was previously stripped and preserved following [24]. The mating scheme was based on an incomplete factorial design, with four groups of five dams mated to four groups of eight sires, with a sire to dam mating ratio of 8 to 5. All parents were NNV-free. Eggs were mixed and distributed in four rearing tanks by ensuring equal representation of dams and sires within each tank. At 164 d post-hatching, fish were transferred to a sea cage, where they were reared until the time of the experimental challenge test.

### Stress test and VNN challenge test

At an age of 539 d post-hatching, 652 randomly chosen experimental fish were transferred from the sea cage to the Istituto Zooprofilattico Sperimentale delle Venezie (IZSVe, Legnaro, PD) and distributed into three close-system 2500-L tanks plus three close-system 380-L tanks for an adaptation period of 9 d. Each tank was filled with artificial salt water (30‰ salinity, temperature 21 ± 1 °C, and oxygen 6 ppm) and exposed to an artificial photoperiod of 10 h of light and 14 h of darkness. Fish were fed with a commercial diet (MRF Marine 3P, Skretting). After the adaptation period, fasting fish were divided into 37 batches of 17 individuals and one batch of 23 individuals; one group was immediately subjected to an acute stress induced by confinement at high density (~ 80 kg/m^3^, 17 fish in a 0.03 m^3^ tank, 23 fish in a 0.04 m^3^ tank) for 10 to 14 min. The 17 fish were caught using a separate mobile enclosure that was put inside the rearing tank. When a sufficient number of fish had swum into the enclosure, it was closed, the fish were caught, and transferred to the tank for stress testing. This avoided the remaining fish inside the rearing tank being stressed due to the fishing operations. After the stress test, fish were anesthetized with 30 ppm of MS-222, weighed (body weight at 548 d post-hatching), blood-sampled from the caudal vein, and then individually tagged.

For infection, we used the virus strain RGNNV 283.2009 that was isolated from animals belonging to a commercial stock during a severe outbreak, which had occurred in the northern Adriatic Sea and caused a 35% mortality rate in sea bass following experimental infection by immersion [8]. The strain RGNNV 283.2009 was propagated on a clone of the SSN1 cell line [4] and the titer was assessed by endpoint dilution assays. Infection was performed by intramuscular injection of 0.1 mL of viral suspension (RGNNV 283.2009, batch 7/16; 10^8.30^ TCID_50_ per mL). After the infection, fish were put into a new tank that was identical in size and characteristics to the tank in which they had been reared during the adaptation period.

At the beginning of the VNN challenge test, temperature was increased to 25 ± 1 °C, while salinity was maintained at 30‰ and oxygen at 6 ppm. The photoperiod and diet were the same as in the pre-test period. Fish were checked three times a day to identify typical behaviours and clinical signs of VNN (abnormal swimming patterns, lethargy, skin darkening, loss of appetite) and dead fish were removed each time and recorded as 1. The experiment ended at 29 d post-challenge and all live fish, recorded as 0 (survivors), were euthanized with an overdose of anaesthetic (MS-222).

After collection, blood samples were kept at 4 °C overnight, centrifuged and the serum was transferred to a new tube. All serum samples were stored at − 20 °C until use for the indirect ELISA assay and for cortisol analysis by radioimmunoassay (RIA). Tissue samples (muscle and fin) were collected from both the fish that died during the experiment and the euthanized survivors at the end of the challenge test and preserved in absolute ethanol for subsequent genomic DNA extraction.

The stress test and all subsequent operations (anaesthesia, weighing, blood-sampling and infection) were repeated one batch at a time, until 629 fish were stressed; the last group was constituted by 23 fish, and the water volume was increased from 0.03 m^3^ to 0.04 m^3^ to avoid variations in the parameters of the experimental procedure. All the operations were completed in one day. The experimental protocol was evaluated and approved by the Italian Ministry of Health (Law decree 26/2014 art. 31; permission number: 975/2016-PR of 13/10/2016).

### Indirect ELISA and cortisol measurement assays

Indirect ELISA for antibodies against NNV was performed on the serum collected before the infection at the Istituto Zooprofilattico Sperimentale delle Venezie (IZSVe, Legnaro, PD), following the protocol developed by [25, 26]. Each sample was measured in duplicate wells and optical density values (OD 450 nm) of control wells were automatically subtracted from OD 450 nm sample values. The antibody titer was quantified based on the ELISA OD sample to positive ratio (S/P ratio):

$$S/P\;ratio = \frac{{sample\;mean \left( {mean\;of\;OD} \right) - negative\;control\;mean}}{positive\;control\;mean - negative\;control\;mean}.$$

Serum cortisol was extracted following the protocol described in [27]. Eight mL of diethyl ether were added to 100 µL of serum, shaken, centrifuged and kept at − 20 °C. The supernatant was decanted in a new tube and dried under a stream of nitrogen at 40 °C. Dried samples were reconstituted in 1 mL of phosphate buffer (PBS, pH 7.2) with bovine serum albumin (BSA) 0.1%. Solid-phase radioimmunoassay (RIA) in microplates of the samples was performed as described in [28] with minor modifications. The radioactivity was counted on a β-counter (Top-Count NXT, Perkin Elmer Life and Analytical Sciences) and the assessed radioactivity values were analysed with the GraphPad Prism 5.0 software (La Jolla, CA, USA), which created a calibration curve and provided the cortisol concentration of each sample (pg per well). Cortisol concentration expressed as pg per well was converted to ng per mL of serum.

To validate the RIA cortisol measures, an extractive yield test, a parallelism test, and inter- and intra-assay precision tests were performed. The extractive yield test was performed by adding a fixed quantity of the radioactive tracer solution to randomly chosen samples. The β-counter reading allowed estimation of the percentage of the radioactive tracer solution that was regained after the extraction protocol. The tests of parallelism were achieved in two steps: first, by analysing the serially diluted extracts of two samples with high cortisol concentrations and, second, by analysing two extracted samples with high and low cortisol concentrations that were diluted together, each time using a different quantity of the “high” and the “low” sample. The inter/intra-assay precision test was performed by analysing six repetitions of the same extracted samples from different plates and from the same plate.

### Genomic sequencing, genotyping, and parentage assignment

Genomic DNA (gDNA) was extracted from approximately 20 mg of tissue (muscle or fin) of each fish using the commercial kits Invisorb® Spin Tissue Mini Kit and Invisorb® DNA Tissue HTS 96 Kit (Invitek, STRATEC Biomedical, Germany). Integrity of gDNA was assessed by visualization on a 1% TAE agarose gel stained with SYBR® Safe DNA Gel Stain (Invitrogen—ThermoFisher Scientific). Genomic DNA concentration and purity were quantified with a NanoDrop ND-1000 spectrophotometer (Thermo Fisher Scientific, Waltham, Massachusetts, USA) in terms of absorbance at 260/230 nm and at 260/280 nm. This procedure ensured that similar concentrations of high-quality gDNA were obtained for each fish, as required for 2b-RAD library preparation. A 2b-RAD library was constructed for each individual following the protocol from [29] with minor modifications. The concentration of each purified individual library was quantified using the Qubit® ds DNA BR Assay kit (Invitrogen—ThermoFisher Scientific) and Mx3000P qPCR instrument (Agilent Technologies, Santa Clara, California, USA). Individual libraries were pooled into equimolar amounts (52 for parents, 96–97 for offspring). Pooled libraries were analysed with Agilent 2100 Bioanalyzer (Agilent Technologies, Santa Clara, California, USA) and then sequenced on an Illumina HiSeq4000 platform with a 50 bp single-read module at Fasteris SA (Plan-les-Ouates, Switzerland; http://www.fasteris.com) and UC Davis (http://comailab.genomecenter.ucdavis.edu). To assess the robustness of the methods, two libraries were replicated from two samples [Technical Replicates, (TR)].

Demultiplexing and an initial check of raw data quality were performed by the sequencing services Fasteris SA and UC Davis. The software FastQC (http://bioinformatics.babraham.ac.uk) was used to visualize and confirm the quality of the raw demultiplexed reads. A custom-made script was used to filter the reads for the presence of the restriction endonuclease recognition site and to trim the adaptors used in the process of libraries construction, which resulted in 34-bp fragments ready to be processed by the STACKS software 2.0 [30]. The trimmed reads were first mapped against the European sea bass genome (http://seabass.mpipz.mpg.de/DOWNLOADS/dicLab1_scaffold.fasta) [31] using a length fraction of 1.0 and a similarity fraction equal to 0.9. Mapping results were exported in SAM format and used as input for *refmap_map.pl* in STACKS. First, the program runs *gstacks,* to build loci according to the alignment positions provided for each read before calling SNPs in each sample. The STACKS module *populations* was used to filter the outputs, excluding the loci that were shared by less than 75% of the analysed individuals. SNPs that had a minor allele frequency lower than 1% and a missing genotype in more than 15% of the individuals, and those that deviated from the expected Hardy–Weinberg equilibrium frequencies (*P* < 0.001) were discarded. Missing genotypes for the remaining SNPs were then imputed using the FImpute software [32].

Parentage assignment was performed with the likelihood-based program CERVUS 3.0 [33, 34]. For each individual tested, parentage was either assigned to the most-likely candidate parental pair with a level of confidence of 80% or left unassigned otherwise. The R package sequoia [35] was used to identify sibling relationships between the 52 parents, assigning them dummy parents and reconstructing a high-likelihood pedigree of the experimental fish. The number of iterations of sibship clustering was set to 30, the maximum number of offspring for a single individual to 100, the threshold log_10_-likelihood ratio (LLR) required for accepting a proposed relationship to 0.5, and the threshold LLR used to identify candidate relatives to − 2.

### Statistical analyses

#### Descriptive statistics

Descriptive statistics for the investigated traits were obtained using the MEANS and UNIVARIATE procedures in SAS 9.4 (SAS Institute Inc.). The frequency distribution of serum cortisol concentration, measured as ng per mL of serum, was skewed, and it was normalized by a square root transformation of the original data. The Kaplan–Meier product-limit survival curve of the experimental fish challenged with NNV was estimated using the LIFETEST procedure in SAS 9.4. Preliminary analyses to identify non-genetic effects on traits were performed with logistic regression and linear models, using the LOGISTIC and GLM procedures of SAS 9.4.

#### Genetic parameters

Variance components and genetic parameters were estimated using Bayesian procedures for the statistical models described in the next section. Marginal posterior distributions for (co)variance components and related parameters were estimated using Monte–Carlo Markov chain (MCMC) methods, performing numerical integration via the Gibbs sampler, as implemented in the software TM [36]. The size of the Gibbs chain, the number of initial samples to discard (burn-in) and the thinning interval were chosen on the basis of Raftery and Lewis convergence diagnostic [37] in a set of preliminary analyses. The Geweke convergence diagnostic based on the Z-value criterion [38] was used to check convergence for each Markov chain. A single Gibbs chain of 1,000,000 iterations was generated in each trait analysis. One Gibbs sample was saved every 100 iterations and the initial 1000 samples of the saved Gibbs chain were discarded when the marginal posterior densities of (co)variance components and their parameters were estimated.

#### Univariate genetic parameters

Variance components for mortality coded as 0 (alive) and 1 (dead) were estimated using the following univariate sire-dam threshold model:

$$l_{ijkm} = \mu + t_{i} + s_{j} + d_{k} + e_{ijkm} ,$$

where ${l}_{ijkm}$ is a latent unobservable variable (liability), $\mu$ is the model intercept, ${t}_{i}$ is the fixed effect of the challenge test tank $i$, ${s}_{j}$ is the random additive genetic effect of sire $j$, ${d}_{k}$ is the random additive genetic effect of dam $k$, and ${e}_{ijkm}$ is a random residual. Sire and dam effects were assumed to be uncorrelated. A threshold model properly handles the dichotomous nature of the trait mortality [36, 39] by using a probit link function to model the latent unobservable variable liability, and connect it to the observed phenotype (0: alive, 1: dead), which depends on whether the underlying liability is lower or higher than a specific threshold. For identification purposes, the residual variance was set to 1 and the threshold to 0. In the Gibbs sampler for threshold models, liability is considered as a nuisance parameter and integrated out in the Gibbs sampler. At each iteration, for each binary phenotype record (0 = alive, 1 = dead), a liability is generated below or beyond the threshold such that the observed phenotype is 0 or 1. Liability was sampled within the bounds -999 and + 999.

Variance components for body weight, cortisol concentrations and antibody titer were estimated using the following univariate animal model:

$$y_{i} = \mu + a_{i} + e_{i} ,$$

where ${y}_{i}$ is a phenotypic record, $\mu$ is the model intercept, ${a}_{i}$ is the random additive genetic effect of the animal, and ${e}_{i}$ is the random residual effect. Additive genetic relationships between animals used in this model were computed from the pedigree, which included all challenged animals, their parents and dummy grandparents.

In all Bayesian analyses, the a priori distribution for fixed effects was a uniform distribution, while the vectors of random effects were assumed normally distributed with a mean zero and variance equal to $\mathbf{I}{\sigma }_{s}^{2}$, ${\mathbf{I}\sigma }_{d}^{2}$, $\mathbf{A}{\sigma }_{a}^{2}$ and ${\mathbf{I}\sigma }_{e}^{2}$ for sire, dam, animal and residual effects, respectively, where $\mathbf{A}$ is the numerator relationship matrix between animals, and $\mathbf{I}$ is the identity matrix. Scaled inverted χ^2^ distributions were assumed as prior distributions for the sire, dam, additive genetic and residual variance. The hyperparameters of scaled inverted χ^2^ distributions ($v$ and ${s}^{2}$) were equal to − 2 and 0, making the prior distribution flat. For each sample of the Gibbs chains, heritability was computed as ${h}^{2}=\left[2\left({\sigma }_{s}^{2}+{\sigma }_{d}^{2}\right)\right]/{\sigma }_{p}^{2}$ for the sire-dam model, where ${\sigma }_{s}^{2}$ and ${\sigma }_{d}^{2}$ are the sire and dam components of the variance, respectively, and ${\sigma }_{p}^{2}$ is the phenotypic variance, and as ${h}^{2}={\sigma }_{a}^{2}/{\sigma }_{p}^{2}$ for the animal model, where ${\sigma }_{a}^{2}$ is the additive genetic component of variance [40]. The median of the marginal posterior density was used as a point estimate for variance components and heritability. The probability for ${h}^{2}$ of being greater than 0.1 (critical threshold to identify a low heritability trait) and the bounds of the 95% highest posterior density interval (HPD95%) [41], which reflects the uncertainty in the estimated parameter, were obtained from the estimated posterior density of ${h}^{2}$ using the R package BOA [42].

#### Bivariate genetic parameters

Bivariate Bayesian analyses were used to estimate (co)variance components and to investigate the relationships (genetic and phenotypic) between the traits, using the same models as used in the univariate analyses. When mortality was one of the analysed traits, a bivariate sire-dam model was used, otherwise a bivariate animal model was fitted to the data. Prior densities for the model effects were those described for univariate analyses. Inverse Wishart prior distributions, which are very vague priors, were used as priors for all (co)variances.

For the bivariate sire-dam models, genetic (${r}_{a}$) and phenotypic (${r}_{p}$) correlations were computed as:

$$r_{a} = \frac{{\left[ {2(cov_{{s\left( {1,2} \right)}} + cov_{{d\left( {1,2} \right)}} ) } \right]}}{{\sqrt {\left[ {2\left( {\sigma_{s\left( 1 \right)}^{2} + \sigma_{d\left( 1 \right)}^{2} } \right)} \right]\left[ {2\left( {\sigma_{s\left( 2 \right)}^{2} + \sigma_{d\left( 2 \right)}^{2} } \right)} \right]} }},$$

$$r_{p} = \frac{{\left( {cov_{{s\left( {1,2} \right)}} + cov_{{d\left( {1,2} \right)}} + cov_{{e\left( {1,2} \right)}} } \right)}}{{\sqrt {\sigma_{p\left( 1 \right)}^{2} \sigma_{p\left( 2 \right)}^{2} } }},$$

where ${cov}_{s(\mathrm{1,2})}$, ${cov}_{d(\mathrm{1,2})}$ and ${cov}_{e(\mathrm{1,2})}$ are the sire, the dam and the residual components of the covariance between traits 1 and 2, respectively, ${\sigma }_{s}^{2}$ and ${\sigma }_{d}^{2}$ are the sire and dam variance, respectively, and ${\sigma }_{p}^{2}$ is the phenotypic variance (computed as ${\sigma }_{s}^{2}+ {\sigma }_{d}^{2}+ {\sigma }_{e}^{2}$) [40].

For bivariate animal models, correlations were estimated as:

$$r_{a} = \frac{{cov_{{a\left( {1,2} \right)}} }}{{\sqrt {\sigma_{a\left( 1 \right)}^{2} \sigma_{a\left( 2 \right)}^{2} } }},$$

$$r_{p} = \frac{{\left( {cov_{{a\left( {1,2} \right)}} + cov_{{e\left( {1,2} \right)}} } \right)}}{{\sqrt {\sigma_{p\left( 1 \right)}^{2} \sigma_{p\left( 2 \right)}^{2} } }},$$

where ${cov}_{a(\mathrm{1,2})}$ and ${cov}_{e(\mathrm{1,2})}$ are the additive genetic and residual components of the covariance between traits 1 and 2, respectively, ${\sigma }_{a}^{2}$ is the additive genetic variance, and ${\sigma }_{p}^{2}$ is the phenotypic variance (computed as ${\sigma }_{a}^{2}+ {\sigma }_{e}^{2}$) [40].

Similar to the univariate analyses, the median of the estimated posterior densities of (co)variances, ${h}^{2}$, ${r}_{a}$, and ${r}_{p}$ was used as a point estimate of the parameters. When the point estimate of a correlation (genetic or phenotypic) was positive (negative), the posterior probability for the correlation of being greater than zero (lower than zero) was computed. Inferences on antagonism or synergy between traits were based on the computed probabilities.

## Results

### Nervous necrosis virus challenge test

**Fig. 1:**
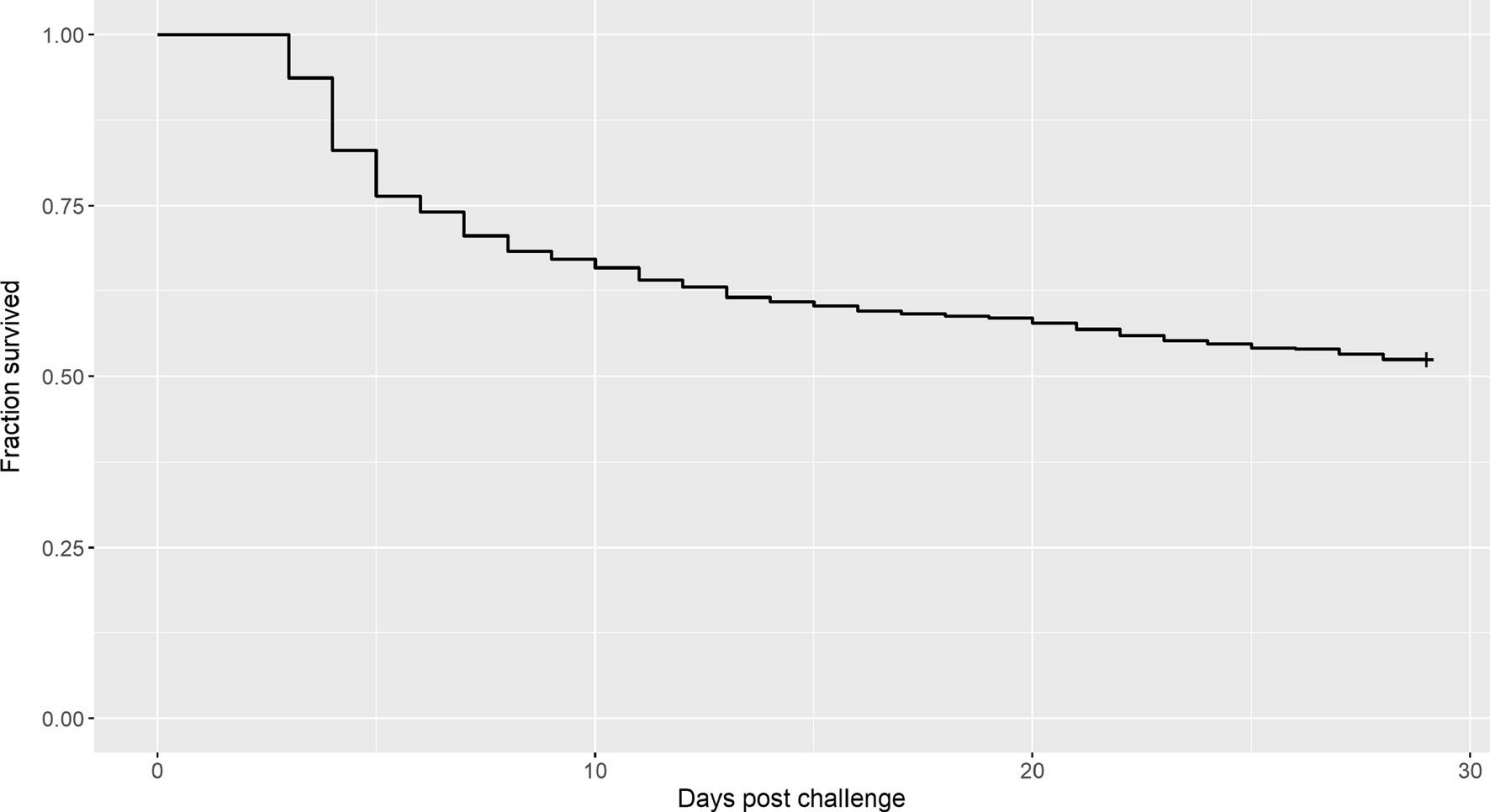
Estimated Kaplan–Meier curve for survival of fish during the challenge test with NNV

Typical clinical signs related to NNV infection were detected in each experimental tank starting day 3 after injection. As illustrated by the estimated Kaplan–Meier curve (Fig. 1), mortality reached a peak at day 4 post-challenge, sharply decreased up to day 9 post-challenge, and then decreased slowly. The overall survival rate at the end of the test was 52.2% across tanks and 10.5, 63.2, 73.3, 50.7, 30.6, and 66.3% in tanks 1 to 6.

### SNP discovery, genotyping, parentage assignment and pedigree reconstruction

In total, 340 million demultiplexed and filtered-quality reads were obtained for the 714 samples (including 12 TR). The number of reads per individual was on average 3,500,000. STACKS identified 351,203 loci (or tags) at 12,018,544 sites (253,999 variant sites). After filtering, the dataset consisted of 18,097 SNPs. Some SNPs were discarded based on a minor allele frequency lower than 1%, genotype frequencies deviating from Hardy–Weinberg equilibrium, and a missing genotype rate higher than 15%. After imputation, the final number of available SNP genotypes per animal was 16,075. Two individuals of the challenged group were discarded from the dataset because of poor genotyping results.

Parentage assignment to a unique parental pair was achieved for 100% of the experimental fish. A total of 41 parents were assigned to 22 dummy parental pairs (14 grandsires and 8 granddams), 3 to 3 single dummy parents (1 grandsire and 2 granddams), while 8 parents remained unassigned. In total, 136 full-sib families were identified among the fish that were challenged with NNV, with the number of offspring per family ranging from 1 to 17 (on average 4.8 full-sibs per family). Two males and one female of the parent group used to generate the experimental fish were not assigned to any individual in the challenged sample. The number of offspring per sire ranged from 4 to 45 (on average 21 offspring per sire), while the number of offspring per dam ranged from 10 to 68 (on average 34 offspring per dam).

### Descriptive statistics

Descriptive statistics for body weight (g) at 548 d post-hatching, post-stress cortisol concentration in blood serum (ng/mL), the square root of cortisol concentration (ng^0.5^/mL^0.5^), and of antibody titer are in Table 1. The body weight of experimental fish at 548 days of age was variable. The average level of cortisol in the serum of the fish assessed after acute stress was high and highly variable across individuals. Likewise, variation in antibody titer was large. In addition to normalization of the frequency distribution, the square root transformation of cortisol concentration decreased the variation in the data.

**Table 1:** Descriptive statistics for the investigated traits

Trait	Mean	CV	Minimum	Maximum
BW (g)	146.42	29.18	35.00	318.00
HC (ng/mL)	213.26	56.86	7.65	855.10
SRHC (ng^0.5^/mL^0.5^)	13.97	30.34	2.76	29.24
AT	74.92	41.52	7.91	173.53

*BW* body weight at 548 days post-hatching, *HC* serum cortisol concentration, *SRHC* square root transformation of HC, *AT* antibody titer: sample to positive ratio of the optical density values (OD 450 nm)

### Univariate genetic parameters

Mortality had the lowest estimated heritability (0.15; Table 2). The probability for the heritability to be greater than 0.1 was 0.70 for mortality. The estimate of heritability was moderate for cortisol concentration (0.19), intermediate for antibody titer (0.36), and high for body weight (0.57), with probabilities of being higher than the critical value of 0.1 greater than 93% (Table 2). The HPD95% intervals of the estimated heritabilities were heterogeneous and wider for body weight and antibody titer than for mortality and cortisol concentration (Table 2).

**Table 2:** Univariate estimates of additive genetic variance (${\sigma }_{a}^{2}$) and heritability (${h}^{2}$) for the investigated traits

Trait	$^{a}{\sigma }_{a}^{2}$	$^{a}{h}^{2}$	*P* (${h}^{2}$> 0.1)	HPD95%
MORT	0.146	0.14	0.697	0.016, 0.307
BW	1198	0.57	1.000	0.331, 0.838
SRHC	3.421	0.19	0.933	0.065, 0.342
AT	372	0.36	0.999	0.163, 0.592

*MORT* post-challenge mortality (0: alive, 1: dead), *BW* body weight (g) at 548 days post-hatching, *SRHC* square root of serum cortisol concentration (ng^0.5^/ml^0.5^), *AT* antibody titer (sample to positive ratio of the optical density values, *OD* 450 nm)

^a^The median of the marginal posterior density has been used as a point estimate of the parameter

*P* ($${h}^{2}$$> 0.1): probability of $${h}^{2}$$> 0.1

### Bivariate genetic parameters

Estimates of heritability for mortality using the bivariate model were consistently higher than those obtained with the univariate model, but similar between the two models for body weight, cortisol concentration, and antibody titer (Table 3). The phenotypic correlations between the investigated traits were not statistically different from zero, except for the correlation between antibody titer and body weight (0.24), for which the probability of having a positive correlation was higher than 95% (Table 3).

**Table 3:** Estimates^a^ of heritabilities and correlations obtained in the bivariate analyses

Trait 1	Trait 2	Trait 1			Trait 2			Correlations		
		${\sigma }_{a}^{2}$	${\sigma }_{p}^{2}$	${h}^{2}$(HPD95%	${\sigma }_{a}^{2}$	${\sigma }_{p}^{2}$	${h}^{2}$(HPD95%	${r}_{p}$(HPD95%)	${r}_{a}$(HPD95%)	*P*
MORT	BW	0.26	1.13	0.228 (0.057, 0.468)	879	1953	0.451 (0.245, 0.702)	− 0.085 (− 0.215, 0.044)	− 0.388 (− 0.878, 0.198)	0.884
MORT	SHRC	0.25	1.13	0.223 (0.058, 0.455)	4.5	19.2	0.237 (0.078, 0.449)	0.020 (− 0.107, 0.148)	− 0.078 (− 0.746, 0.625)	0.578
MORT	AT	0.27	1.13	0.236 (0.063, 0.467)	336	1030	0.328 (0.158, 0.539)	− 0.022 (− 0.149, 0.099)	− 0.388 (− 0.866, 0.191)	0.886
SHRC	AT	3.6	18.6	0.196 (0.068, 0.366)	424	1079	0.394 (0.189, 0.635)	− 0.035 (− 0.136, 0.070)	0.115 (− 0.420, 0.631)	0.815
SHRC	BW	3.6	18.6	0.193 (0.066, 0.358)	1268	2148	0.592 (0.354, 0.851)	− 0.023 (− 0.134, 0.090)	0.268 (− 0.275, 0.773)	0.652
AT	BW	410	1069	0.384 (0.175, 0.631)	1290	2160	0.599 (0.361, 0.859)	0.249 (0.134, 0.366)	0.490 (0.085, 0.814)	1.000

*MORT* post-challenge VNN mortality as a binary trait (0: alive, 1: dead), *BW* body weight (g) at 548 d post-hatching, *SRHC* square root of serum cortisol concentration (ng^0.5^/ml^0.5^), *AT* antibody titer (sample to positive ratio of the optical density values, OD 450 nm)

^a^The median of the marginal posterior density has been used as a point estimate of parameters; (co)variance components were estimated with bivariate sire-dam models when mortality was one of the analysed traits or with bivariate animal models when mortality was not considered. A probit model was used for mortality. ${\sigma }_{a}^{2}$: additive genetic variance; ${\sigma }_{p}^{2}$: phenotypic variance; ${r}_{p}$: phenotypic correlation; ${r}_{a}$: additive genetic correlation

Uncertainty in the estimates of genetic correlations was large, resulting in large 95% HPD intervals (Table 3). Estimates of genetic correlations between mortality and body weight or antibody titer were moderately negative (− 0.39), with a probability of being lower than zero equal to 88%. The estimate of the genetic correlation between cortisol concentration and antibody titer was moderate (0.27), with a probability of having a positive correlation equal to 81%. Body weight and antibody titer exhibited a positive genetic correlation estimate (0.49), while body weight and cortisol concentration were genetically uncorrelated (Table 3).

## Discussion

Interest in selective breeding as a tool to enhance genetic resistance to VNN in European sea bass has markedly increased in recent years, because of the major threat represented by NNV for sea bass hatcheries and aquaculture farms, combined with the lack of effective therapeutical remedies for this disease [1, 43]. The presence of a genetic basis for resistance to various diseases in fish species, which is evidenced by moderate to high estimates of heritability, suggests that selective breeding can be an effective tool to reduce disease outbreaks or to limit negative consequences of such outbreaks [1, 44]. In addition to direct approaches, in which relatives of the selection candidates are challenged with NNV, selective breeding can exploit physiological (e.g., post-stress cortisol levels) or immunological (e.g., antibody titer) indicator traits that exhibit significant additive genetic variation and non-zero genetic correlations with VNN resistance.

### Genetic variation in VNN mortality

In this study, genetic parameters for mortality after VNN challenge of a sea bass experimental population and genetic correlations between mortality and potential indicator traits (body weight, antibody titer and cortisol concentration after stress exposure) were estimated. The heritability of mortality on the liability scale, estimated using a univariate sire-dam model, was low in comparison to estimates obtained in other studies [14, 15]. Differences with literature estimates can be attributed to differences between the studies, such as the type of broodstock, the mating design used to generate the experimental fish, the number of fish challenged, the method of infection, and size of the challenged fish. In comparison with our study, Doan et al. [14] challenged 1420 fish at an average body weight of 16.3 g, that were produced by a full factorial mating design involving sires and dams captured from four wild populations. Palaiokostas et al. [15] used a sample of 1538 juveniles that were generated by sires and dams of a commercial breeding strain, that were infected by immersion at an average body weight of 10 g. When information on other traits (body weight, antibody titer, and cortisol concentration) was exploited in the estimation process, using bivariate models, our estimate of heritability for VNN mortality increased and the differences with estimates reported in studies that used larger samples, different broodstocks, and smaller fish [14, 15] were smaller.

In our study, age (548 d post-hatching) and size of the experimental fish at the time of infection were rather unusual compared with those of previous investigations on sea bass [14, 15] or other species [45–48]. Frequently, challenge tests are performed using young juveniles (at 200 d post-hatching and a body weight of 10 to 20 g). Infectivity of the nodavirus is very high in sea bass larvae and juveniles and mortality rates can reach 100% [6]. However, our study confirms that, even in advanced juveniles, VNN can have significant consequences, which have been described only in one previous paper, on a natural VNN natural outbreak [7]. This is particularly important, as it means that fish that have almost reached harvesting stage can be severely affected by VNN, which represents a serious cost for aquaculture farms, both in terms of animal product loss and investments.

The use of small fish in challenge tests is preferred when the infection is carried out by placing the animals in a water environment that contains the pathogen. Such experimental trials based on infection by immersion closely mimic the conditions of natural infections, where pathogens must cross the external protective barriers of the host (physical or mechanical barriers, epithelium, and mucus), penetrate the host cell barriers in adequate numbers, attack target cells, and replicate. Conversely, when animals are infected by injection of the pathogen, the trial is less representative in terms of natural disease outbreaks, but the advantages are the application of a systematic infection, with an equal level of exposure to the virus for all individuals, and greater effectiveness when large-sized individuals are tested [49]. An equal level of exposure is very important when the major aim of a challenge test is to compare resistance across families, such as in genetic studies or when evaluating selection candidates. Regardless, the choice of infection method is largely arbitrary and infection by intraperitoneal injection is fairly common across studies, even when the size of the tested animals is small [14, 45]. Only in one study on VNN resistance in sea bass [15] were animals infected by immersion.

We can hypothesize that different methods to infect the animals lead to different results, particularly when the focus of the investigation is on genetic variation of disease resistance. The comparison between genetic studies in which experimental infections were carried out with different modalities (immersion *vs*. injection) is not straightforward because, with injection, only part of the mechanisms of resistance is considered, i.e. the components of specific immunity (lymphocytes, and antibodies) and those of the non-specific immune system related to physical, chemical and cellular defences (such as granulocytes and macrophages, complement system, and inflammation). Variation in resistance due to differences in the characteristics of the external barriers (skin, and mucous membranes) is ignored with injection, which may lead to a decrease in the observed variability in resistance and affect heritability. With infection by immersion, physical and mechanical barriers can play a significant role in preventing penetration of the virus and contribute additional variation to the observed resistance.

### Relationships between body weight and VNN mortality

Mortality and body weight were genetically correlated, with a posterior probability of being negative equal to 0.88. This implies that selection programs that aim at increasing body weight at a fixed age or daily gain in sea bass strains may yield a favourable correlated response in resistance to VNN. Our results are, however, not consistent with those of [14], who reported an unfavourable genetic correlation between body weight at 180 d post-hatching and resistance to VNN. This inconsistency may be due to size differences of the infected fish, the quantity of viral copies injected, survival rate after infection, and the use of a different statistical methodology to estimate (co)variance components. Compared to our study, Doan et al. [14] infected fish of smaller size, obtained a survival rate higher than 70% at the end of the challenge test, and estimated the genetic correlation using a linear model for survival instead of a probit model, which is the model of choice for binary traits. The estimate of the genetic correlation reported in [14] also had a large standard error, which indicates a large uncertainty about the estimate. Although the uncertainty associated with estimates of genetic correlations between traits was also large in our study, as indicated by the size of the HPD95% intervals, the posterior probability for the genetic correlation between mortality and body weight to be negative was high.

In our study, NNV experimental infection was by injection of an equal dose of viral suspension, with no dose adjustment for size of the fish. This may raise questions about the efficacy of the dose used, since the variation in body weight across individuals was large. In particular, we questioned whether an equal dose of injection could have caused a greater across-family variability in infection rate and greater survival in families that had greater genetic potential for growth, which could contribute to the negative estimate of the genetic correlation between mortality and body weight. However, the estimate of the phenotypic correlation between mortality and body weight was negligible, which contradicts a possible role of the dose used on differences in mortality between families.

To simplify experimental procedures, sex identification was not performed in our study, which means that we could not account for possible variation arising from sex effects in the estimation of heritability. Not accounting for sex effects may have increased the estimate of the residual variation, to some extent, and, therefore, reduced estimates of heritability. One question that could be raised is whether the sexual dimorphism in size (female sea bass are significantly larger than males [50]), may have affected the results of the challenge test, with a greater survival in females because of their greater weight. This is, however, contradicted by the trivial estimate of the phenotypic correlation between mortality and body weight obtained in our study.

### Genetic parameters of post-stress cortisol concentration

Heritability estimates for post-stress cortisol concentration were moderate and showed a posterior probability of being higher than 0.1 greater than 90%. These heritability estimates are in the range of those reported in the literature for sea bass [20, 21]. Post-stress cortisol concentration was not correlated with VNN mortality, either genetically or phenotypically. Thus, the effectiveness of selecting breeding candidates on EBV for post-stress cortisol concentration as an indirect strategy to enhance resistance to VNN of sea bass is questionable. Estimates of genetic correlations between cortisol concentration and VNN mortality for sea bass have not been reported in the literature. However, negligible correlations have been estimated for vibriosis resistance in Atlantic cod [51], which are consistent with estimates in our study. Other studies [52–55] on other species (rainbow trout, salmon and carp) have provided inconsistent results about differences in disease resistance between strains selected for high and low cortisol concentration.

### Genetic parameters of antibody titer against NNV

The heritability of antibody titer against NNV had a point estimate equal to 0.36 and a probability of being greater than 0.1 close to 1. This estimate was higher than estimates reported for salmonids [22, 23], which ranged from 0.02 to 0.18, and indicates the presence of a genetic basis for this trait in sea bass. Antibody titer was negatively genetically correlated with mortality, which suggests that antibody titer may play a role as an indicator trait of resistance to VNN and that selection of individuals with high EBV for antibody titer results in a favourable correlated response for resistance to VNN. Challenge tests on relatives of breeding candidates or to generate data for genomic selection can benefit from the measurement of antibody titers on both fish that died and that survived during the test. It might be interesting to extrapolate these results to antibody titers resulting from vaccination. However, variation in antibody titers from a NNV challenge is affected by a large group of host-related mechanisms (external barriers and specific and non-specific immunity), while vaccination only results in variation that mirrors the efficiency of the specific immune system. In addition, immunogenicity of the vaccine is commonly enhanced by the addition of adjuvants [56].

### New indicator traits and genomic selection as future perspectives

Other possible indicator traits for disease resistance can also be exploited in indirect selection approaches. In general, nodavirus infection appears to be cooperatively prevented by activation of innate immunity (NK cells and antimicrobial peptides), cellular T cell type I interferon immunity, and humoral immunity (immunoglobulins antibodies) [57, 58]. In the literature, several immunological parameters have been proposed as useful traits to identify resistant animals. They are mainly components of the innate, or non-specific, immune system, such as serum lysozyme, myeloperoxidase, respiratory burst, complement activity, haemolytic and bactericidal activities [59], and natural antibodies [60, 61]. However, for some of these traits, additive genetic variation has been estimated to be small, which makes their exploitation as selection criteria in a breeding program difficult or ineffective [59, 62]. Since the genetic parameters of innate immune system components are poorly studied, additional scientific efforts may be directed to specific studies.

Other future perspectives for selective breeding for disease resistance could be the exploitation of genomic selection as an alternative to traditional pedigree-based methods [63]. Genomic selection can help overcome difficulties related to individual phenotyping for some of the traits analysed in this study, such as VNN mortality and antibody titer. The construction of 2b-RAD libraries, sequencing, SNP discovery, and genotyping introduced in this study were performed in a broader view of development of genomic predictions for selection candidates for the traits of interest, and not only for parentage assignment and pedigree reconstruction.

## Conclusions

Identification of a genetic basis for mortality, body weight, cortisol concentration, and antibody titer suggests that selective breeding can be an effective approach to enhance these traits in European sea bass and should be integrated in the pool of VNN prevention tools for farmed sea bass. Antibody titer against NNV offers the opportunity of indirect selection approaches to enhance resistance to VNN through the exploitation of its negative genetic correlation with mortality. Traditional pedigree-based breeding programs require individual phenotyping for mortality and antibody titer, which may be difficult or impossible for routine implementation. Such difficulties can be overcome by developing genomic tools for the prediction of the genetic merit of selection candidates for the traits of interest. In this view, the effectiveness and accuracy of genomic prediction of the genetic merit of selection candidates for VNN mortality and antibody titer deserve specific investigations. Studies are also required to elucidate the effects of the various infection methods that are currently used in challenge tests (immersion *vs* injection) on the observed variation in resistance to VNN and its additive genetic component.

## Data Availability

The data supporting the findings of this study are available from Valle Cà Zuliani Società Agricola srl, but restrictions apply to the availability of these data, which were used under license for the current study and are not publicly available. Data are however available from the authors upon reasonable request and with permission of Valle Cà Zuliani Società Agricola srl.

## References

[1] Doan QK, Vandeputte M, Chatain B, Morin T, Allal F (2016). Viral encephalopathy and retinopathy in aquaculture: a review. J Fish Dis.

[2] Mori KI, Nakai T, Muroga K, Arimot M, Mushiake K, Furusawa I (1992). Properties of a new virus belonging to nodaviridae found in larval striped jack (*Pseudocaranx dentex*) with nervous necrosis. Virology.

[3] Munday BL, Nakai T (1997). Nodaviruses as pathogens in larval and juvenile marine finfish. World J Microbiol Biotechnol.

[4] Iwamoto T, Nakai T, Mori K, Arimoto M, Furusawa I (2000). Cloning of the fish cell line SSN-1 for piscine nodaviruses. Dis Aquat Organ.

[5] Volpe E, Gustinelli A, Caffara M, Errani F, Quaglio F, Fioravantia ML, Ciulli S (2020). Viral nervous necrosis outbreaks caused by the RGNNV/SJNNV reassortant betanodavirus in gilthead sea bream (*Sparus aurata*) and European sea bass (*Dicentrarchus labrax*). Aquaculture.

[6] Munday BL, Kwang J, Moody N (2002). Betanodavirus infections of teleost fish: a review. J Fish Dis.

[7] Le Breton A, Grisez L, Sweetman J, Ollevier F (1997). Viral nervous necrosis (VNN) associated with mass mortalities in cage-reared sea-bass, *Dicentrarchus labrax* (L.). J Fish Dis.

[8] Vendramin N, Toffan A, Mancin M, Cappellozza E, Panzarin V, Bovo G (2014). Comparative pathogenicity study of ten different betanodavirus strains in experimentally infected European sea bass*, Dicentrarchus labrax* (L.). J Fish Dis.

[9] Coeurdacier JL, Laporte F, Pepin JF (2003). Preliminary approach to find synthetic peptides from nodavirus capsid potentially protective against sea bass viral encephalopathy and retinopathy. Fish Shellfish Immunol.

[10] Thiéry R, Cozien J, Cabon J, Lamour F, Baud M, Schneemann A (2006). Induction of a protective immune response against viral nervous necrosis in the European sea bass *Dicentrarchus labrax* by using Betanodavirus virus-like particles. J Virol.

[11] Nuñez-Ortiz N, Pascoli F, Picchietti S, Buonocore F, Bernini C, Toson M (2016). A formalin-inactivated immunogen against viral encephalopathy and retinopathy (VER) disease in European sea bass (*Dicentrarchus labrax*): immunological and protection effects. Vet Res.

[12] Valero Y, Awad E, Buonocore F, Arizcun M, Esteban MA, Meseguer J (2016). An oral chitosan DNA vaccine against nodavirus improves transcription of cell-mediated cytotoxicity and interferon genes in European sea bass juveniles gut and survival upon infection. Dev Comp Immunol.

[13] Gonzalez-Silvera D, Guardiola FA, Espinosa C, Chaves-Pozo E, Esteban MA, Cuesta A (2019). Recombinant nodavirus vaccine produced in bacteria and administered without purification elicits humoral immunity and protects European sea bass against infection. Fish Shellfish Immunol.

[14] Doan QK, Vandeputte M, Chatain B, Haffray P, Vergnet A, Breuil G (2017). Genetic variation of resistance to Viral Nervous Necrosis and genetic correlations with production traits in wild populations of the European sea bass (*Dicentrarchus labrax*). Aquaculture.

[15] Palaiokostas C, Cariou S, Bestin A, Bruantn JS, Haffray P, Morin T (2018). Genome-wide association and genomic prediction of resistance to viral nervous necrosis in European sea bass (*Dicentrarchus labrax*) using RAD sequencing. Genet Sel Evol.

[16] Das S, Sahoo PK (2014). Markers for selection of disease resistance in fish: a review. Aquacult Int.

[17] Dorson M, Quillet E, Hollebecq MG, Torhy C, Chevassus B (1995). Selection of rainbow trout resistant to viral haemorrhagic septicaemia virus and transmission of resistance by gynogenesis. Vet Res.

[18] Storset A, Strand C, Wetten M, Kjøglum S, Ramstad A (2007). Response to selection for resistance against infectious pancreatic necrosis in Atlantic salmon (*Salmo salar* L.). Aquaculture.

[19] Pottinger TG, Branson EJ (2008). The stress response in fish—mechanisms, effects and measurement. Fish Welfare.

[20] Volckaert FAM, Hellemans B, Batargias C, Louro B, Massault C, Van Houdt JKJ (2012). Heritability of cortisol response to confinement stress in European sea bass *Dicentrarchus labrax*. Genet Sel Evol.

[21] Vandeputte M, Porte JD, Auperin B, Dupont-Nivet M, Vergnet A, Valotaire C (2016). Quantitative genetic variation for post-stress cortisol and swimming performance in growth-selected and control populations of European sea bass (*Dicentrarchus labrax*). Aquaculture.

[22] Strømsheim A, Eide DM, Hofgaard PO, Larsen HJS, Refstie T, Røed KH (1994). Genetic variation in the humoral immune response against *Vibrio salmonicida* and in antibody titre against *Vibrio anguillarum* and total IgM in Atlantic salmon (*Salmo salar*). Vet Immunol Immunopathol.

[23] Fjalestad KT, Larsen HJS, Røed KH (1996). Antibody response in Atlantic salmon (*Salmo salar*) against *Vibrio anguillarum* and *Vibrio salmonicida* O-antigens: heritabilities, genetic correlations and correlations with survival. Aquaculture.

[24] Fauvel C, Boryshpolets S, Cosson J, Wilson Leedy JG, Labbé C, Haffray P (2012). Improvement of chilled seabass sperm conservation using a cell culture medium. J Appl Ichthyol.

[25] Scapigliati G, Buonocore F, Randelli E, Casani D, Meloni S, Zarletti G (2010). Cellular and molecular immune responses of the sea bass (*Dicentrarchus labrax*) experimentally infected with betanodavirus. Fish Shellfish Immunol.

[26] Nuñez-Ortiz N, Stocchi V, Toffan A, Pascoli F, Sood N, Buonocore F (2016). Quantitative immunoenzymatic detection of viral encephalopathy and retinopathy virus (betanodavirus) in sea bass *Dicentrarchus labrax*. J Fish Dis.

[27] Bertotto D, Poltronieri C, Negrato E, Majolini D, Radaelli G, Simontacchi C (2010). Alternative matrices for cortisol measurement in fish. Aquacult Res.

[28] Simontacchi C, Bongioni G, Ferasin L, Bono G. Messa a punto di un metodo RIA su micropiastra per il dosaggio diretto del progesterone ematico. Atti XLIX Convegno Nazionale S.I.S.Vet., 1995. p. 343–4.

[29] Wang S, Meyer E, McKay JK, Matz MV (2012). 2b-RAD: a simple and flexible method for genome-wide genotyping. Nat Methods.

[30] Catchen J, Hohenlohe PA, Bassham S, Amores A, Cresko WA (2013). Stacks: an analysis tool set for population genomics. Mol Ecol.

[31] Tine M, Kuhl H, Gagnaire PA, Louro B, Desmarais E, Martins RST (2014). The European sea bass genome and its variation provide insight into adaptation to euryhalinity and marine speciation. Nat Commun.

[32] Sargolzaei M, Chesnais JP, Shenkel FS (2014). A new approach for efficient genotype imputation using information from relatives. BMC Genomics.

[33] Marshall TC, Slate J, Kruuk LEB, Pemberton JM (1998). Statistical confidence for likelihood-based paternity inference in natural populations. Mol Ecol.

[34] Kalinowski ST, Taper ML, Marshall TC (2007). Revising how the computer program CERVUS accommodates genotyping error increases success in paternity assignment. Mol Ecol.

[35] Huisman J (2017). Pedigree reconstruction from SNP data: parentage assignment, sibship clustering and beyond. Mol Ecol Res.

[36] Legarra A, Varona L, López de Maturana E. TM Threshold model; 2008. http://snp.toulouse.inra.fr/~alegarra/. Accessed 01 June 2018.

[37] Raftery AE, Lewis SM, Bernardo JM, Berger JO, Dawid AP, Smith AF (1992). How many iterations in the Gibbs Sampler?. Bayesian statistics.

[38] Geweke J, Bernardo JM, Berger JO, Dawid AP, Smith AF (1992). Evaluating the accuracy of sampling-based approaches to the calculation of posterior moments (with discussion). Bayesian statistics.

[39] Sorensen D, Gianola D (2002). Likelihood, Bayesian, and MCMC methods in quantitative genetics.

[40] Falconer DS, Mackay TFC (1996). Introduction to quantitative genetics.

[41] Chen MH, Shao QM (1999). Monte Carlo estimation of Bayesian credible and HPD intervals. J Comput Graph Stat.

[42] Smith BJ (2007). boa: an R Package for MCMC output convergence assessment and posterior inference. J Stat Softw.

[43] Buonocore F, Nuñez-Ortiz N, Picchietti S, Randelli E, Stocchi V, Guerra L (2019). Vaccination and immune responses of European sea bass (*Dicentrarchus labrax* L.) against betanodavirus. Fish Shellfish Immunol..

[44] Ødegård J, Baranski M, Gjerde B, Gjedrem T (2011). Methodology for genetic evaluation of disease resistance in aquaculture species: challenges and future prospects. Aquac Res.

[45] Bangera R, Ødegård J, Nielsen HM, Gjøen HM, Mortensen A (2013). Genetic analysis of vibriosis and viral nervous necrosis resistance in Atlantic cod (*Gadus morhua* L.) using a cure model. J Anim Sci..

[46] Henryon M, Berg P, Olesen NJ, Kjær TE, Slierendrecht WJ, Jokumsen A (2005). Selective breeding provides an approach to increase resistance of rainbow trout (*Onchorhynchus mykiss*) to the diseases, enteric redmouth disease, rainbow trout fry syndrome, and viral haemorrhagic septicaemia. Aquaculture.

[47] Vallejo R, Palti Y, Liu S, Evenhuis J, Gao G, Rexroad C (2014). Detection of QTL in rainbow trout affecting survival when challenged with *Flavobacterium psychrophilum*. Mar Biotechnol (NY).

[48] Guy D, Bishop SC, Woolliams JA, Brotherstone S (2009). Genetic parameters for resistance to infectious pancreatic necrosis in pedigreed Atlantic salmon (*Salmo salar*) post-smolts using a Reduced Animal Model. Aquaculture.

[49] Robinson NA, Gjedrem T, Quillet E, Jeney G (2017). Testing for resistance: natural outbreaks versus controlled challenge testing. Fish disease, prevention and control strategies.

[50] Saillant E, Fostier A, Menu B, Haffray P, Chatain B (2001). Sexual growth dimorphism in sea bass *Dicentrarchus labrax*. Aquaculture.

[51] Kettunen Præbel A, Westgård JI, Peruzzi S, Fevolden SE (2007). Genetic parameters for post-stress cortisol activity and vibriosis resistance in Atlantic cod (*Gadus morhua* L.). Aquaculture.

[52] Fevolden SE, Refstie T, Røed KH (1992). Disease resistance in rainbow trout (*Oncorhynchus mykiss*) selected for stress response. Aquaculture.

[53] Fevolden SE, Røed KH (1993). Cortisol and immune characteristics in rainbow trout (*Oncorhynchus mykiss*) selected for high or low tolerance to stress. J Fish Biol.

[54] Fevolden SE, Nordmo R, Refstie T, Røed KH (1993). Disease resistance in Atlantic salmon (*Salmo salar*) selected for high or low responses to stress. Aquaculture.

[55] Yin Z, Lam T, Sin Y (1995). The effects of crowding stress on the non-specific immunoresponse in fancy carp (*Cyprinus carpio* L.). Fish Shellfish Immunol..

[56] Plant KP, La Patra S (2011). Advances in fish vaccine delivery. Dev Comp Immunol.

[57] Chen YM, Wang TY, Chen TY (2014). Immunity to betanodavirus infections of marine fish. Dev Comp Immunol.

[58] Costa JZ, Thompson KD (2016). Understanding the interaction between Betanodavirus and its host for the development of prophylactic measures for viral encephalopathy and retinopathy. Fish Shellfish Immunol.

[59] Magnadóttir B (2006). Innate immunity of fish (overview). Fish Shellfish Immunol.

[60] Parmentier H, Lammers A, Hoekman J, Reilingh G, Zaanen I, Savelkoul H (2004). Different levels of natural antibodies in chickens divergently selected for specific antibody responses. Dev Comp Immunol.

[61] Wondmeneh E, Van Arendonk J, Van der Waaij E, Ducro B, Parmentier H (2015). High natural antibody titers of of indigenous chickens are related with increased hazard in confinement. Poult Sci.

[62] Mohanty B, Sahoo P (2007). Edwardsiellosis in fish: a brief review. J Biosci.

[63] Robledo D, Palaiokostas C, Bargelloni L, Martínez P, Houston R (2017). Applications of genotyping by sequencing in aquaculture breeding and genetics. Rev Aquac.

